# Access to HIV care in jails: Perspectives from people living with HIV in North Carolina

**DOI:** 10.1371/journal.pone.0262882

**Published:** 2022-01-24

**Authors:** Colleen Blue, Mara Buchbinder, Mersedes E. Brown, Steve Bradley-Bull, David L. Rosen

**Affiliations:** 1 Institute for Global Health and Infectious Diseases, University of North Carolina at Chapel Hill, Chapel Hill, North Carolina, United States of America; 2 Department of Social Medicine, Center for Bioethics, University of North Carolina at Chapel Hill, Chapel Hill, North Carolina, United States of America; 3 Division of Infectious Diseases, Department of Medicine, University of North Carolina at Chapel Hill, Chapel Hill, North Carolina, United States of America; Geneva University Hospitals, SWITZERLAND

## Abstract

Most incarcerations for people living with HIV (PLWH) occur in jails, yet studies of HIV care during jail incarceration are limited. As part of a larger study to explore the ethical considerations in extending public health HIV surveillance to jail settings, we conducted semi-structured interviews with twenty-three PLWH with more than 300 distinct jail incarcerations post HIV diagnosis in 21 unique North Carolina jails. Interviews included questions about HIV disclosure in jail, the type of HIV care received in jail, and overall experiences with HIV care in jail. We report on participants’ experiences and perspectives in four domains: access to HIV care in jail; impact of jail incarceration on continuity of HIV care; privacy and stigma; and satisfaction with HIV care in jail. Although most participants received HIV medications and saw providers while in jail, almost half reported that their greatest challenge in regard to HIV care was obtaining their HIV medications in the face of limited jail resources or policies that made access to medications difficult. Findings from this study suggest that jail leadership should review internal policies regarding HIV medications to ensure that PLWH can receive them quickly upon entry into jail. Findings also suggest that more external resources are needed, for example from state and local health departments, so that jails can provide timely HIV medications for PLWH incarcerated in their facilities.

## Introduction

There are 1.2 million people living with HIV (PLWH) in the United States, and each year, one in seven passes through correctional settings [1]. Periods of incarceration can disrupt continuity of HIV care and impact the ability to maintain adherence to antiretroviral therapy (ART) and achieve viral suppression [2–5]. At the same time, incarceration can provide an opportunity to re-engage PLWH in care and optimize healthcare [6, 7].

Although most incarcerations in the US—including those for PLWH—occur in jails [1], most research examining HIV care during incarceration has been conducted in prisons. Yet, the HIV treatment challenges in these two settings can differ. Jails are usually locally resourced and managed and hold individuals awaiting trial, parole, or serving sentences of less than one year [8]. Prisons, on the other hand, are under the jurisdiction of the state or federal government and hold individuals sentenced to more than one year [9]. As such, jails tend to have fewer healthcare resources than prisons, and jail incarcerations typically last a few days or weeks [8], as compared to prison stays, which are typically much longer [9]. Studies including jails typically have been set in unified systems (i.e., in states which combine their prison and jails systems) or in massive urban jails which are unrepresentative of most of the 3200 jails across the US [10]. In addition, prisons and jails are often conflated in research, making it difficult to parse findings specific to jails.

Much of the limited research about the impact of jail incarceration on HIV care focuses on short-term and long-term linkages to care post jail release [11–13]. However, research in the prison context suggests that PLWH may face significant challenges in accessing HIV care while incarcerated in jails. Access to HIV healthcare resources, including specialists and medications, vary within and across prison systems, affecting the care received [14]. Considering that jails are in most cases managed independently, at the county level, the quality and availability of healthcare across these facilities are likely to be much more heterogenous than in prisons.

Concerns about privacy and stigma in incarcerated settings may also affect the willingness of PLWH to disclose an HIV diagnosis to healthcare providers, and in turn, may diminish access to care in jails [15]. In contrast to prison [16], universal or opt-out HIV testing in most jails is uncommon [17, 18]; therefore, access to HIV care in jail depends on individuals’ self-disclosure of HIV status to jail healthcare providers. Correctional officers often mediate communication between incarcerated individuals and healthcare staff, and their relationships with incarcerated individuals can influence an individual’s comfort in disclosing their status and when they receive care [19, 20]. Fear of discrimination by correctional officers or other incarcerated people can result in delayed disclosure or in not disclosing one’s status at all to healthcare providers. Some incarcerated people in both jail and prison settings have expressed concerns about involuntary disclosure of HIV-status to peers and correctional officers through gossip, perceived stereotypes, or medication distribution [21, 22]. Fears of physical harm, alienation, and the inability to engage in supportive and protective relationships with other incarcerated people because of disclosure can override the desire to access care [19].

Jail incarceration raises unique concerns about access to HIV care as compared to prison incarceration. For these reasons, jail incarceration may have a distinct impact on HIV care. However, relatively little is known about access to HIV care in jail and how privacy and stigma concerns impact HIV care in jail settings. As part of a larger study to explore the ethical considerations in extending public health HIV surveillance to jail settings, the goal of this sub-study was to examine PLWH’s experiences accessing HIV care during jail incarcerations.

## Methods

### Study population

The study protocol was approved by the Wake County Human Services Institutional Review Board, the North Carolina Department of Public Safety, and the Institutional Review Board of the University of North Carolina at Chapel Hill. Written informed consent was obtained by all research participants. From March 2019 to March 2020, we conducted semi-structured qualitative interviews with PLWH formerly incarcerated in a North Carolina jail to understand the ethical and practical considerations of using publicly available jail records, court records, and state health department HIV diagnosis records to improve the continuity of care for PLWH previously incarcerated in jails in North Carolina [23]. As part of this larger study, we conducted a sub-study to understand the experiences of PLWH accessing HIV care in North Carolina jails. At the time of the interviews, participants were either living in the community or in a prison facility. Interviews included a series of questions focused on personal experiences with HIV care in jail settings. Interviewers used a semi-structured interview guide that included questions focused on HIV disclosure in jail, the type of HIV care received during jail incarcerations, and overall experiences with HIV care in jail (See S1 and S2 Appendixes). Participants had to meet the following eligibility criteria: HIV positive, 18 years old or older, English speaking, and either transferred from jail to prison or released from jail into the community in the last six months. The study protocol was approved by the Wake County Human Services Institutional Review Board, the North Carolina Department of Public Safety, and the Institutional Review Board of the University of North Carolina at Chapel Hill. More extensive details about the research design, methods, and analysis have been reported elsewhere [23].

#### Community sample: Recruitment and data collection

Participants were recruited in the community via partnerships with a local HIV case management organization and a local health department’s HIV clinic. The project manager screened each participant over the phone for eligibility and then set up a time for interviewers to meet with the participant. Interviews were conducted by two study staff members who had training and previous experiences interviewing incarcerated and formerly incarcerated populations. Prior to the start of each interview, interviewers explained the study and made sure each participant understood that participation was voluntary and how their information would be protected. Each community interview was audio recorded and later transcribed by a professional transcription service. Interviews took place in a private office space with the door closed at the local HIV case management organization, with only the interviewer and interviewee present. Community participants received a $35 gift card for participation.

#### Prison sample: Recruitment and data collection

Recruitment took place at two different prison facilities, one for men and one for women. Participants were recruited with assistance from a prison infectious disease provider who worked at both facilities. Because audio recorders were not permitted in prison, one interviewer conducted the interview and the other took notes and wrote down direct quotes when possible. After the interview was completed, the interviewers discussed and added any additional relevant details to the written summary.

Prison participants did not receive remuneration in accordance with prison policy and to minimize risk of coercion. In addition to the consent procedure described above for community-based participants, there was additional discussion with prison participants about the lack of incentives available for their participation, including no effect on length of their prison sentence or living conditions in prison, and assurance that none of the information given would be shared with the prison system. Individuals who chose to participate did not receive any differential treatment as compared to participants who declined. Prison and community-based interviewees were both told that they could opt out of participation at any time for any reason, without penalty. Each interview was conducted in a private office space located in the prison medical clinic with the door closed and only the interviewer, notetaker, and participant present. Prison medical clinic and security personnel were nearby in case of any adverse event, and study staff had completed training from prison personnel regarding safety within the prison environment. Moreover, study staff had successfully conducted hundreds of prison interviews for previous studies without incident.

### Data analysis

After audio-recorded interviews (community sample) were transcribed verbatim, transcripts and summaries (prison sample) were analyzed using Dedoose qualitative analysis software. [24] The research team identified a set of 24 inductively generated thematic codes, and codes were assigned to relevant portions of the transcript or summary. Analytic procedures are described more thoroughly elsewhere [23]. After coding was completed, we reviewed relevant coding reports to identify salient sub-themes and patterns for further analysis. This article focuses on codes pertaining to disclosure, stigma, privacy, HIV care in jails, and transitions in and out of jail.

## Results

Twenty-four PLWH recently incarcerated in jail consented to participate in a qualitative interview. One participant was eliminated from this sub-study analysis because she was diagnosed with HIV after being incarcerated in jail during her current prison stay. Therefore, the final sample for this article includes 23 participants, including 10 participants from the community and 13 participants from the prison sites. Most participants were male (n = 16), Black (n = 18), and ages 45–54 (n = 10). Most participants had been living with HIV for more than 10 years (n = 16) and many had more than five jail stays post-diagnosis (n = 11) (See Table 1). Participants reported more than 300 distinct jail incarcerations post-HIV diagnosis in 21 unique North Carolina county jails. A few people with high numbers of jail incarcerations post-diagnosis had to estimate their response. Below, we describe participants’ experiences and perspectives in four key domains: (1) access to HIV care in jail; (2) impact of jail incarceration on continuity of HIV care; (3) privacy and stigma; and (4) satisfaction with HIV care in jail.

**Table 1:** Participant demographics.

Characteristic	Community n = 10 (%)	Prison n = 13 (%)	Total n = 23 (%)
**Sex**			
Female	4 (40%)	1 (7.7%)	5 (21.7%)
Male	4 (40%)	12 (92.3%)	16 (69.6%)
Transgender (m->f))	2 (20%)	0 (0%)	2 (8.7%)
**Age**			
18–24	0	1 (7.1%)	1 (4.3%)
25–34	2 (20%)	2 (15.4%)	4 (17.4%)
35–44	3 (30%)	2 (15.4%)	5 (21.7%)
45–54	5 (50%)	5 (38.5%)	10 (43.5%)
55–64	0	3 (23.1%)	3 (13%)
**Race/Ethnicity**			
Black/African American	8 (80%)	10 (76.9%)	18 (78.3%)
White	1 (10%)	2 (15.4%)	3 (13%)
Hispanic/Latino	0	0	0
American Indian or Alaska Native	0	1 (7.7%)	1 (4.3%)
Mixed race	1 (10%)	0	1 (4.3%)
**Education**			
Some high school	2 (20%)	0	2 (8.7%)
High school or GED	7 (70%)	9 (69.2%)	16 (69.6%)
Some college	0	2 (15.4%)	2 (8.7%)
Degree from professional, technical, or trade school	1 (10%)	1 (7.7%)	2 (8.7%)
Unknown	0	1 (7.7%)	1 (4.3%)
**Years living with HIV**			
<5	0	2 (15.4%)	2 (8.7%)
5–10	2 (20%)	3 (23.1%)	5 (21.7%)
11–20	5 (50%)	2 (15.4%)	7 (30.4%)
21–30	3 (30%)	6 (46.2%)	9 (39.1%)
**# Jail stays post diagnosis**			
1–5	4 (40%)	8 (61.5%)	12 (52.2%)
6–10	1 (10%)	2 (15.4%)	3 (13%)
More than 10	5 (50%)	3 (23.1%)	8 (34.8%)

### Access to HIV care in jail

#### Medication

Access to medication and corresponding wait times for receiving medication varied by jail incarceration for each individual, but most participants (n = 18) were typically able to access HIV medications while incarcerated in jail, and 17 of these participants typically received HIV medications within one week. At the same time, many (n = 14) also reported periods when they had not taken medication while incarcerated in jails for a variety of reasons that included delays in getting the correct medication, short length of stay, the jail declining to provide medication due to the financial burden, or personal choice. One participant, who did not receive HIV medications during a two-day jail stay, explained, “they [jail staff] didn’t have no time to get your information…take about three days, four days for them to find out who you are and this and that” (community-based participant, 32-year-old man). Another participant, who went without HIV medications during a 10-day jail stay, explained that he did not receive medication when being transferred from one jail to another. Jail staff told him, “You’re supposed to have it [medications] brought it with you. I was like, ‘Well, they [jail staff] didn’t give it to me’” (community-based participant, 41-year-old man).

One participant attributed worse access to HIV medications in jails as compared to prisons to the financial cost: “The county’s not like the state. The state would foot the bill because you’re going to prison. But the county don’t want to foot that bill. Well, they would say that when you got medication at home, we’ll call somebody and tell them to bring it.” Alternatively, he explained, if the patient did not have medications at home, the jail would try to obtain medications at no-cost from the health department. “But the county, they ain’t trying to pay that bill” (community-based participant, 52-year-old man).

Many participants (9/18) reported that they relied on family members to bring HIV medications to the jail rather than the jail providing medications on its own. As one participant explained, “My cousin’s a CO [correctional officer]. And if I see my cousin, I say, ‘Call, my grandma and let her know to bring my wallet,’ and my grandma know when I’m in the cell, bring my meds. Or I always have my pill thing on me. I have at least four doses on me, enough to carry me over until they get me over” (community-based participant, 25-year-old woman). Similarly, another participant reported having learned to have a family member bring in his medication to avoid long delays, by which point “the virus could have mutated” (prison-based participant, 49-year-old man).

The strategy of having a family member bring medications was not always successful. One participant, who reported going without medications during an entire 60-day incarceration, said that he asked jail staff to call his mother and request that she bring in his medication. The jail staff indicated that no one had answered the phone, but according to the participant, “My mom like, ‘ain’t nobody call this phone.’ I don’t think they called. They don’t really care” (community-based participant, 52-year-old man). Another participant noted that he tried to arrange to have his sister bring in medication but was told that that was not permitted (prison-based participant, 59-year-old man).

Six participants reported delays in receiving medications of longer than a week during at least some of their jail stays, which sometimes resulted from jail policies requiring PLWH to see an infectious disease provider or have their medication verified by a pharmacy before continuing with treatment. “The system moves slow,” one participant said. “They don’t care if you take heart medication; you’re not gonna get it [on time]” (community-based participant, 51-year-old man). This participant noted that county jails are slow to get people any medications, not just HIV medications. Another participant reported that there were days during one jail stay that no one showed up with the cart to distribute medication (prison-based participant, 59-year-old man).

#### Providers

Most participants (18/23) stated that they typically saw a nurse or physician for their HIV care during their incarceration. Participants were most frequently transported to a community clinic for the visit (n = 11). Among those who did not see an HIV provider, the primary barrier was cost. Although jails do not typically require that inmates pay to receive routine care for preexisting chronic conditions, most charge a co-pay (~$5-$20) for impromptu “sick call” requests for medical visits that are not routine. One participant explained that he considered the costs in deciding whether “sick call was going to be worth it in the long run” (prison-based participant, 28-year-old man). Furthermore, he maintained, “Being diagnosed with something, you shouldn’t have to pay to be seen.” Another participant indicated that jail staff had failed to make an appointment: “I just sit there waiting, waiting, waiting, waiting. Thinking that they made the appointment … and they never did” (community-based participant, 29-year-old trans woman).

Among those who did see HIV providers, interactions were generally described favorably. As one participant said: “They [medical staff] treat you good,” (community-based participant, 25-year-old woman). Another participant emphasized that the nurse and physician tried to improve care and connect people with local resources, but that the jail administrators would not support HIV care (prison-based participant, 28-year-old man).

#### Non-medical resources

Some participants also reported that HIV care could be hampered by lack of access to non-medical resources necessary for HIV management. One participant chose not to take her medication in jail because the jail did not provide sufficient food to combat the medication’s gastrointestinal side effects. These side effects caused by HIV medications often required her to have immediate access to a restroom, which was unavailable to her in jail. She explained, “At [name of jail], you don’t have a bathroom in your room. You have to keep buzzing and ringing the buzzer to go to the bathroom. That was a bit much, but still, I just didn’t want to take it because of the side effects. They were horrifying” (community-based participant, 48-year-old woman). Another participant, who waited weeks to receive his HIV medications upon jail entry, explained that county jails are simply limited in the type of medical care that they can provide. “Jail isn’t equipped to handle people in wheelchairs, cancer, HIV or other people like that,” he stated (prison-based participant, 49-year-old man).

Several participants suggested that the lack of care and empathy among some jail staff interfered with care and sometimes delayed access to vital HIV care and treatment for incarcerated PLWH. One participant said, “You have to jump through hoops…they [detention officers] think we are just liars and do stuff just for the hell of it…They think we don’t get sick” (3009, 54-year-old man). Another explained, “Now in jail, they’ll let you die,” (community-based participant, 52-year-old man). In one of the more extreme examples, a participant described not receiving HIV medications for the entire duration of a two-month jail stay “because jail staff did not bring it.” After crying repeatedly, a jail staff member told her not to worry because “it would not turn into AIDS overnight” (prison-based participant, 54-year-old woman). Several participants (n = 14) mentioned non-medical resources that helped them manage HIV in jail, such as self-discipline regarding taking medications, advocating for oneself among medical staff, belonging to a supportive religious community, jail medical staff’s knowledge of HIV status without patient disclosure, having outside support from local AIDS services organizations to assist with making appointments in the jail, and family members advocating for them to receive HIV treatment.

### Impact of jail incarceration on continuity of HIV care

The impact of jail incarceration on continuity of HIV care varied among participants. For some, jail incarceration represented a disruption in their HIV care, for others there was no impact, and for a small group their jail stay(s) sometimes had a positive impact. Almost half of the participants (9/21) indicated that their time in jail negatively affected their HIV care, mostly due to missing or not receiving HIV medications during their incarceration. One participant explained that because he missed HIV medications while in jail, his “viral load was up…but it is now undetectable [in prison]” (prison-based participant, 28-year-old man). Several individuals (8/21) indicated that their jail stays had no effect on their continuity of care because they were able to access the care they needed, or their jail stay was too short to have an impact. For example, one participant described his experience with HIV care in jail as sufficient, saying, “I mean every jail I ever been to, they’ve always taken me to doctors’ appointments” (community-based participant, 51-year-old man).

Four participants who expressed mixed experiences with HIV care in jail concluded that some of their jail stays ultimately had a positive impact on their HIV care by providing them with an opportunity to focus on their health. Three of them explained that jail incarceration helped support their access to medications and sobriety without social distractions that exist outside of the jail environment:

Getting locked up [in jail] really helped me get back on track with my medication because like I said, I had been out in the world…drinking, and drugging, and anything but the medication (community-based participant, 41-year-old man).

Probably saved my life plenty of times, being incarcerated [because I] had a clear head (prison-based participant, 54-year-old man).

It just helped me because when I found out I had [HIV], I went to jail right after that…if I would have stayed [on the outside] …I would have been worse off (prison-based participant, 53-year-old man).

Nevertheless, two participants explained that having a jail stay changed the way their doctor outside of jail viewed them. One described how her doctor was “upset” with her for continually going to jail (community-based participant, 35-year-old woman), and another explained, “My doctor… she wasn’t judgmental, but…I feel like [time in jail] impacted how she looked at me” (community-based participant, 48-year-old woman).

### Privacy and stigma

Most participants (15/23) chose to keep their HIV status private from detention staff. Participants’ reasons for nondisclosure included that jail officers may be misinformed about how HIV is transmitted, may treat PLWH differently, or may disclose their status to others. One participant explained that if officers find out someone’s HIV status, they can use it against them by telling other officers or incarcerated people, explaining “[HIV status] isn’t confidential in jail,” (prison-based participant, 49-year-old man). Another participant said that he did not share his status with jail officers because he “didn’t know who [officers] knew on the outside,” who might also know the participant (prison-based participant, 42-year-old man). A third participant explained that she kept her status private from detention staff because “the staff gossip more than inmates…they let out more information than they should,” (prison-based participant, 54-year-old woman). This participant described how, after learning her status, jail staff avoided touching her cutlery and prohibited her from passing out food trays. She stated that if she complained about not getting her medication or how she was treated, she was often sent to “the hole [restrictive housing, also known as solitary confinement].”

In addition to participants who kept their status private from jail staff due to concerns about possible negative consequences, three participants described keeping their HIV status private from detention staff simply because they preferred to keep their information private. As one participant put it: “I mean, [my HIV status] ain’t none of their [detention officers’] business first of all” (community-based participant, 32-year-old man). Another explained, “[HIV status is] really something you really don’t like share [with] people, you know, besides somebody in your family” (community-based participant, 52-year-old man). On the other hand, several participants (n = 7) mentioned that they occasionally disclosed their status to officers to get medications, to ensure that staff would know their status in case of a medical emergency, or because they knew which officers they could trust to keep it private. One participant explained, “I would let most officers know [my status] so in case if I did have a seizure, they knew how to respond,” (community-based participant, 48-year-old woman).

Most participants (20/23) kept their HIV status private from other incarcerated individuals in jail to avoid stigma and violence and to maintain privacy. Many (n = 18) explained that privacy is important because the jail setting is particularly conducive to stigma and violence against PLWH. As one participant explained, “‘you got people in there with various charges…And they looking to blame it on somebody else…And so, you know, you don’t want to make yourself a target” (community-based participant, 51-year-old man). Likewise, another participant said, “It’s something you don’t want…people are childish and immature in settings like that” (prison-based participant, 22-year-old man). One participant described how she kept her status private from other people incarcerated in jail “because I didn’t want people to talk and try to look at me as a bad person. I was scared” (community-based participant, 37-year-old trans woman). Another said she did not share her status “so I always be safe” (community-based participant, 29-year-old trans woman). One participant reported that he had not disclosed his HIV status, yet he was often questioned by others incarcerated in jail who were curious about why he had so many medical appointments and about his medications “‘cause they may wanna trade [medications] with you, you know what I mean?” (community-based participant, 32-year-old man).

Despite these concerns, most participants (14/18) reported that they were able to navigate their incarceration and keep their HIV status confidential. For example, one woman described how she would “act out” to get her own cell so that she could maintain privacy around HIV care (prison-based participant, 54-year-old woman). Another participant mentioned that while he initially had concerns about his privacy during incarcerations soon after his diagnosis, more recently he had been able to maintain his privacy by simply not disclosing. “I mean after…you see the process and go through it, then it’s like, "Well… the only people that would know [your HIV status] is if you tell somebody" (community-based participant, 51-year-old man). Participants also reported that the discreet distribution of medications by jail staff helped to maintain their privacy.

Among the four participants who reported that privacy and stigma concerns directly affected their HIV care, one man explained that he had opted not to disclose his HIV status or take medications during an early jail stay because he was young and scared, though he had done so more recently (prison-based participant, 42-year-old man). He described ongoing challenges sharing information with medical staff in jail due to the proximity of officers during medical visits: “Medical stuff is difficult but with officers there, there is no privacy. They [detention officers] are right there and makes it hard to be honest with medical staff. I don’t want to share it [health information].” Another participant reported that he did not feel comfortable sharing details about his health during one of his jail stays because the jail was so small that he “didn’t have privacy…inmates are listening and stuff” (prison-based participant, 42-year-old man). Ultimately, this participant decided to disclose his status, yet he reported that he never received HIV medications, which he perceived to be due to the jail’s financial constraints.

### Satisfaction with HIV care in jail

When asked to assess how satisfied they were with the HIV care that they received in jail, almost half of the participants (11/23) expressed dissatisfaction, mainly due to missing or not receiving HIV medications or concerns about privacy and stigma. Nevertheless, a minority of participants (7/23) were satisfied with the care they received in jail, largely due to receiving HIV medications and attending medical appointments while in jail. “I was always pretty well satisfied and felt they reached the needs of my condition,” said one participant (prison-based participant, 22-year-old man). Although he did not like being shackled when taken to outside appointments, another participant explained that “[jail staff] did an excellent job…I got a chance to go out and get my medications like I was supposed to…I can’t complain, it was good” (prison-based participant, 53-year-old man). Five participants expressed ambivalence or mixed feelings about the care they received in jail either because they received medications but no medical care, or because they had positive experiences in one jail but negative experiences in another.

Notably, participants’ overall assessments of HIV care in jail did not appear to be related to the number of times jailed or to their incarceration in specific jails. For example, among the 11 participants that had been incarcerated in the same jail, four were dissatisfied with the HIV care they received in jail, four were ambivalent or had mixed feelings, and three were satisfied. Similarly, other participants who had incarcerations in the same jail reported different levels of satisfaction with their care. At the same time, participants with a history of multiple incarcerations oftentimes did not distinguish their experiences by jail, impeding our ability to fully understand whether people incarcerated in the same jail had similar experiences or not.

## Discussion

This is one of the few studies to focus on the experiences of PLWH accessing HIV care during jail incarceration as opposed to during prison incarceration or the post-release period. In addition, this is one of the few qualitative studies of HIV care in jail that includes participants from multiple jail facilities. Participants in this study had experienced incarcerations in over one-fifth (21/95) of all county jails in the state, representing jails from diverse geographic regions and with varied resources, suggesting that these interviews reflect a wide array of jail experiences.

Almost half of participants in this study reported that their greatest challenge in regard to HIV care was obtaining their HIV medications in the face of limited jail resources or policies which made access to medications difficult. Despite jails’ legal responsibility to provide and pay for HIV medications [25], many participants had to resort to arranging to have their medications delivered to them by family or friends, and even this tactic was not universally successful. Other studies, which were largely conducted in massive urban jails or unified jail-prison systems, found that the greatest challenges to HIV care during jail incarcerations are maintaining privacy and navigating perceived or enacted stigma [14, 19, 22]. In contrast to those studies, our findings may reflect challenges common to rural or suburban areas, which may not share characteristics with the massive urban jails or unified prison-jail systems featured in correctional health research [6, 10, 13].

Privacy and stigma against PLWH in jail were also significant concerns for most of our participants, as other studies have found. Nevertheless, most participants reported that they were able to keep their HIV status confidential from detention officers and other incarcerated people while in jail, and that their privacy-related concerns did not ultimately affect their HIV care. Only four participants reported that privacy and stigma concerns negatively impacted their HIV care, and for one of them, the jail’s resource limitations had a larger impact on his care. Other challenges mentioned by participants included not seeing an HIV provider while in jail due to cost and a lack of non-medical resources, such as a restroom or empathy among detention officers. These findings suggest that eliminating copayments for doctor visits for PLWH and improving availability of non-medical resources could have a positive impact on HIV management in jail.

Despite the reported challenges, a small group of participants suggested that jail incarceration had a positive impact on their HIV care and general health because incarceration provided them with access to medications and forced period of sobriety. The finding that incarceration, for some people, can improve HIV management and health has been documented previously [6, 19, 26]. Considering the multitude of possible detrimental consequences of incarceration (e.g., loss of employment and housing, family separations), the experiences of this population raise questions about how best to intervene so that HIV and substance use services are more readily accessed in the community, potentially avoiding further incarcerations.

One possible intervention, termed Data-to-Care (D2C), is a collaborative effort in which HIV surveillance data (e.g., Medicaid records, HIV clinical laboratories, and Ryan White Care records) are used to identify PLWH who are not in care, so that their HIV providers or special health department outreach workers contact the patients and facilitate their re-reengagement with community-based HIV care [27]. As part of the re-linkage process, patient needs are assessed and efforts are made to address barriers to HIV care, such as substance use or transportation. Expansion of community D2C services has the potential to preempt or at least alleviate jails’ roles as *de facto* HIV safety net providers. Additionally, our previous research suggests that PLWH and other stakeholders (e.g., HIV providers, privacy experts, community advocates) generally support extending D2C services to jails to help ensure that PLWH who do become incarcerated have access to HIV services during incarceration and after release [28].

This study has several limitations. First, participants included PLWH who had been recently incarcerated in jails in North Carolina; the findings may not be generalizable to jails nationwide. Second, while our sample reflects the demographics of PLWH in North Carolina [29] and people incarcerated in jails [30]. we were unable to recruit a balanced sample of men and women due to restrictions on face-to-face research generated by the COVID-19 pandemic. Our interviews with women as well as transgender individuals suggest that these groups may have distinct experiences when accessing HIV care in jail, suggesting the need for further research of the gendered aspects of incarceration experiences. Finally, we recruited participants from a local case management organization, HIV clinic, and prison infectious disease clinics, locations where PLWH were receiving HIV care or services. Thus, our study is missing the perspective of PLWH who were not currently engaged in HIV care and, by extension, may not have been engaged in care during their time spent in jail. Despite this limitation, we also adopted this recruitment strategy anticipating that most of our participants would have multiple incarcerations, and while they were all currently in care, some were not in care previously, and thus were able to speak to the perspectives of those not in care.

## Conclusion

Participants’ experiences in jail—and the impact of those incarcerations on HIV care and subsequent satisfaction with care—varied considerably. The diverse experiences conveyed in our interviews reflect not only the role of jails’ resources and environments in shaping HIV care, but also the importance of patients’ individual circumstances. Although most participants reported receiving HIV medications and seeing providers while in jail, many also reported significant challenges accessing HIV care in jail. Findings from this study suggest that jail leadership should review internal policies regarding HIV medications to ensure that PLWH can receive them quickly upon entry into jail. Findings also suggest that more external resources are needed, for example from state and local health departments, so that jails can provide timely HIV medications for PLWH incarcerated in their facilities. More research is needed to understand how to improve access to HIV care in jails, especially among people incarcerated in rural and suburban jails. Moreover, the breadth of experiences reported by participants in this study underscores the value of qualitative research to give voice to this too-often marginalized population.

## Supporting information

S1 Appendix Interview guide for community sample. (DOCX)

S2 Appendix Interview guide for prison sample. (DOCX)
