# Supplementary material for: Access to HIV care in jails: Perspectives from people living with HIV in North Carolina
Source: PLoS One. 2022 Jan 24;17(1):e0262882. doi: 10.1371/journal.pone.0262882 (PMC8786150; doi:10.1371/journal.pone.0262882)
Supplement: S2 Appendix — (DOCX) [file pone.0262882.s002.docx]

**S2 Appendix. Interview guide for prison sample.**

Thank you for taking the time to talk with me. The purpose of this project is to learn about HIV care for people who become incarcerated in jail.  We are talking to jail administrators, people who have been incarcerated, and others in the community.  We are asking about people's experiences and their reaction to some new strategies to improve HIV care among people who have been in jail. You are being asked to be in the study because you were recently in jail and have an HIV infection.

I’ll start by asking you questions about your experiences living with HIV and times in your life when you’ve been in jail. Then I’ll talk about the project I work with, which is interested in combining information from the state health department and from jail websites, to help make HIV care better for people in jails.

I’ll be asking you lots of questions. There are no wrong answers here. We are simply interested in learning about your opinions, experiences, and beliefs.

Do you have any questions before we begin?

1. **HIV care before, during, and after jail**

First, I’m going to ask you some questions about times you’ve been in jail, and what was going on with your HIV care during those times. Creating a timeline can help me understand when things occurred, so if you don’t mind, we can create a timeline together. I know that it might be difficult to remember some of the information that I’ll be asking about. That’s okay -- your best guess is still helpful.

1. Timeline questions:
   1. When were you born? [Write the year of the participant’s birth on the left of the timeline.]
   2. When were you diagnosed with HIV? [Write the date of the participant’s diagnosis on the timeline.
   3. Now, I’d like ask about times when you were incarcerated in jail or prison. Let’s start with prison, and I will mark each of them on the timeline here. [Write the start date and length of incarceration on the timeline for each prison stay after the diagnosis date. For prison stays before diagnosis, write the number of times only.]
   4. Now please tell me about the times when you were in jail. Let’s start with the times that you went from a city or county jail directly to prison, and I will mark each of them on the timeline here. Now please tell me about other times when you were incarcerated in a city or county jail, and I will mark each of them on the timeline. [Write the start date and length of incarceration on the timeline for each jail stay after the diagnosis date. For jail stays before diagnosis, write the number of times only.] For jail stays after your diagnosis, can you tell me which jails these were? [Write the jail name for each jail stay post-diagnosis.]

Now I’m going to ask you some questions about your **HIV care outside of jail or prison**.

People with HIV may have times when they see their doctor regularly and get all their medications, and other times when they don’t see their doctor as much as they need, or aren’t able to get their HIV medications.

1. When not in jail or prison, how have things gone for you since you were diagnosed with HIV? (Probe: Seeing an HIV doctor? Getting medications?)
2. What are some of the things that have helped you get the medical care you needed? Be healthy?
3. What are some of the things that have made it difficult to get the medical care you needed? Difficult to be healthy?

Now I’m going to ask you some questions about your time in jail.

When in jail, there are a few groups of people who might learn if an inmate has HIV. This might include jail medical staff, jail officers, or other inmates.

1. During the time(s) when you’ve been in jail, have you ever kept your HIV status private from those working at the jail or from other inmates.

Jail staff Yes  No

Other Inmates Yes  No

Can you tell me about that? What might have happened if others had found out that you’re living with HIV? Probe for: other inmates, jail officers, jail administrators, medical staff.

1. Have you, or anyone you know, ever been treated badly in jail because of having HIV?

Yes  No

Would you mind telling me about that?

1. In general, what kind of medical care have you received for your HIV while in jail?
   1. Have you typically seen a doctor or nurse for your HIV? Yes  No
   2. Have you typically taken HIV medication in jail? Yes  No
      - If you were diagnosed prior to entering jail, how long between entering jail and receiving your medications?

_______________________

- - - If you were diagnosed in jail, how long between being tested for HIV and receiving your medications?

_______________________

- - - Were they the same medications you had taken on the outside?

Yes  No

- - - How were your medications provided to you?
      - Probe: Did you keep the medications yourself or get them at a medication window or cart?
      - Did someone else outside of jail have to bring your medications to you?
    - Did you ever run out of HIV meds when you were in jail? Yes  No
  1. Were there times when you did NOT take HIV medications in jail? Yes  No
     - IF YES: Why didn’t you take medications in jail?
  2. Thinking about the times you’ve been in jail, how satisfied have you been with the amount of care you received?
  3. For times that no care was received: what is the main reason that you didn’t receive any care? In general, have concerns about privacy had an impact on your HIV care? If yes, how?
  4. In general, how did your time in jail affect your HIV care? (Probes: relationship with doctor, medications, viral resistance, health coverage, etc. Probe also for discrepant examples—i.e. good impacts and bad impacts.)

1. In jail, what has typically helped you get the HIV care you need? Be healthy?

In jail, what has typically made it hard for you to get the HIV care you need? Hard to be healthy?

When released from jail, have you typically had any help getting back into HIV care (e.g. seeing your HIV doctor)? Yes  No

Did you ever get any help from jail staff in seeing your doctor in the community? Did you get help from any others? Yes  No

1. Right before going to jail, did you have an HIV doctor? Yes  No
   1. If YES: Did your regular HIV doctor in the community know that you were in jail? If so, how did they find out? Is there anything your regular HIV doctor did, or could have done, to help with your HIV care in jail or afterwards?
2. Were there any times when you were released from jail into the community, as opposed to being transferred to prison? Yes  No
3. If so, how did your HIV care change from inside jail to after release? How long did it take you to get back into care outside of jail? What made that easier/harder?
4. Was there anyone who talked with you before your release about getting HIV care in the community?
5. Were there any times when you went directly from jail to prison? Yes  No
6. If so, how did your HIV care change from jail to prison? Was there a delay in getting your HIV medications after entering prison? How much do you think the prison found out from the jail about your HIV care?
7. During any times when you were in prison, were you on medications? Seeing a doctor regularly? Virally suppressed?
8. **HIV surveillance and Data 2 Care**

Thank you for telling me about your experiences with HIV care in jail. Now I’m interested in your thoughts and experiences with the state health department in North Carolina. As with other diseases that can be passed between people, the state health department keeps track of when someone is diagnosed with HIV. *[Refer participant to Surveillance and Data to Care Diagram.]*

1. A few days after first being diagnosed with HIV, the state health department often sends an outreach worker, also known as a DIS worker, to learn more about how the newly diagnosed person may have been infected and whether s/he may have infected others. Did a state health worker talk to you after you were diagnosed? Yes  No
   1. What was that like? How did they know that you were HIV+?
   2. What, if anything, resulted from your meeting with the outreach worker?
2. Have you had any interactions with any health department outreach workers, also known as bridge counselors, since your initial diagnosis? Yes  No
   1. What were those interactions like?
   2. What, if anything, resulted from your meeting with the outreach worker?

As we’ve been talking about, when someone first tests positive for HIV, the state health department will send an outreach worker to talk to that person. Let's say someone named John Smith tests positive for HIV.  The state health department then sends an outreach worker to talk with John. At the same time, the state health department adds John Smith's name to a list of all HIV-positive people in the state.  After that, whenever John or other HIV + people go to the doctor and have a viral load test, the state health department gets a copy of the results.  If lots of people across the state have a high viral load, the state health department knows that, in general, people aren’t getting as much HIV treatment as they need. Or, when lots of people across the state have a low viral load, the state knows that most people are getting treatment for their HIV.

1. Many people are unfamiliar with this tracking process. How much did you already know about how that works?
2. How do you feel about the state health department keeping track of who tests positive for HIV, and their viral loads?
3. What do you think are the benefits or positive things of the state keeping track of this information? (Probes: for patients? Public? Other entities?)
4. What are the disadvantages or negative things? (Probes: for patients? Public? Other entities?)
5. Do you think the state health department should ask the permission of people living with HIV to keep information about their HIV or other health conditions? Why / why not? If you were given the choice of sharing your information with the state health department, would you share it? Why or why not?

Another thing the health department does is it looks to see who doesn’t have a recent viral load or HIV care appointment, and it sends outreach workers to see if the person needs help getting HIV care.

1. How do you feel about that?
2. Have you ever been contacted by the health department to help you get back into HIV care?

Yes  No

1. What do you think are the benefits or positive things of the state reaching out to people who may not be seeing their HIV doctor? (Probes: for patients? Public? Other entities?)
2. What are the disadvantages or negative things? (Probes: for patients? Public? Other entities?)
3. How might it affect someone if they were accidentally told they were HIV positive when they weren’t?
4. Do you think the state health department should ask the permission of people living with HIV to contact them about HIV care? Yes  No

Why / why not?

1. If a patient is not receiving care, do you think that the state health department should *always* contact the patient to help them get back into care? Yes  No
2. If you were given a choice, would you want the state health department to help you get back into care? Yes  No

***********(*Start Section III at 40 minutes from the beginning of the interview if possible*.)***********

1. **HIV surveillance and Data 2 Care in jails**

Now that we’ve talked about how things are currently done, I want to share what some researchers are discussing to understand HIV in jails. Because many jails have websites where you can find out who is currently incarcerated, the state health department could combine that information with its list of HIV+ persons to find out how many people at each jail have HIV. That way, the state health department would know whether there are people in the jail who need HIV care and they could do things to try to improve care for those folks both during and after their jail stay. [*Refer participant to map.]*

First, information on the number of people with HIV in each jail could be used to **find out which jails need more HIV doctors or more HIV medications.**

1. What are your initial reactions to this idea of combining (jail and health department) data to count the number of people with HIV in jail?
2. What are some *good* things that might happen if the state health department knew the number of HIV+ people in each jail?
3. What are some *good* things that might happen if each county jail knew the number of HIV+ people that it was incarcerating?
4. What are some *bad* things that might happen if the state health department knew the number of HIV+ people in each jail?
5. What are some *bad* things that might happen if each county jail knew the number of HIV+ people that it was incarcerating?

[*Refer participant to comparison list.]* It is also possible that the health department could learn who has HIV in NC jails, and this information could be used to get people the treatment they need while in jail, or to connect them with HIV care after release.

1. What are your initial reactions to this idea? How well do you think this would work?
2. What are some *good* things that might happen if the state health department could learn who has HIV in each jail?
3. What are some *good* things that might happen if each county jail could learn who has HIV in their jail?
4. What are some *bad* things that might happen if the state health department could learn who has HIV in each jail?
5. What are some *bad* things that might happen if each county jail could learn who has HIV in their jail?
6. What would be the best way to get people the care they need while in jail? What would be the best way to connect them with HIV care after release?
   1. Who should make the contact with an out-of-care patient? (Health department? Jail medical staff? Community doctor? Someone else?
   2. When should the contact be made? (In jail? If in jail, where/when? After release?)
   3. Who should be contacted? (Someone who misses HIV appointments for a period of time? Someone who never started HIV care? Never came back for their test results? Has a high viral load? Has no recent lab tests? Other factors?) When should they be contacted?
   4. When people are back in the community, but out of care, how do you think they should be contacted?
   5. What should be done to ensure that people’s HIV status stays confidential?

38. Are there any other groups that might want to use this information? If so, what other groups? How would they use the data? What good or bad things may come of this?

39. Do you think people who aren’t taking HIV medications, or have stopped them, would want help from the health department or jail in getting started on them? Yes  No

Why or why not? How do you feel about using incarceration in jail as an opportunity to get people back on HIV medications?

1. **Information that jails/courts make available to the public**

Thanks for letting me know what you think about this idea. I have just a couple more questions for you about the types of information about people who are in jail and prison that are available to the public. Now I am talking about all people who are incarcerated, not just those who have HIV.

40. To your knowledge, what information about people who are incarcerated **do** jails make available to the public? For people who have been charged, what information **do** courts make available to the public? What information **do** prisons make available to the public?

41. Do you or people you know visit any public (i.e. jail/court/prison) websites to get information about people who are incarcerated? If so, why? How often?

42. How accurate is the information *the jails* have on people who are incarcerated? How accurate is the information *the courts* have on people who are incarcerated? How accurate is the information *the prisons* have on people who are incarcerated? [Probes: correct name, charge, age, address?]

43. Have you ever had any good or bad things happen as a result of your information being made available on these websites?

44. What information about people who are incarcerated **should** jails/courts make available on public websites?

45. Do you think that the state health department should get permission from people before linking their health information with other types of information, like jail records? Yes  No

Why / why not? Would you give your permission?

46. Who should have access to information on who is HIV positive in jails?

- 1. Jail medical staff Yes  No
  2. Jail admin staff Yes  No
  3. State health dept Yes  No
  4. Local health dept Yes  No
  5. Social service agencies Yes  No
  6. Other inmates Yes  No
  7. Other groups/agencies Yes  No

47. What types of legal information, such as arrest charges, court dates, time in jail or prison stays, would you want to keep private from the NC health department? Why?

48. What types of health or medical information would you want to keep private from jails? Why?

49. Is there anything else you’d like to share with us about HIV in jail?

Thank you very much for your time today! We are done.
